# Evolution of morphological traits of *Dendrobium *sensu lato (Orchidaceae)—an attempt to resolve phylogenetic relationships in nominal and morphologically convergent sections

**DOI:** 10.1186/s12870-025-06263-w

**Published:** 2025-02-22

**Authors:** Aleksandra Burzacka-Hinz, Magdalena Dudek, Natalia Olędrzyńska, Aleksandra M. Naczk, Dariusz L. Szlachetko

**Affiliations:** 1 Department of Plant Taxonomy and Nature Conservation, Faculty of Biology, University of Gdańsk, Wita Stwosza 59, Gdańsk, 80308 Poland; 2 Department of Evolutionary Genetics and Biosystematics, Faculty of Biology, University of GdańSk, Wita Stwosza 59, Gdańsk, 80308 Poland

**Keywords:** Dendrobiinae, ITS, Molecular analyses, Morphology, Plastid markers, Taxonomy

## Abstract

**Background:**

*Dendrobium* is a large genus within the Orchidaceae family, containing over 1,600 species. Most are sympodial epiphytes. These species are distributed throughout southern, eastern, and southeastern Asia, the Pacific Islands, Australia, New Guinea, and New Zealand. Infrageneric classification of this group is challenging, largely because of the large number of taxa, the wide geographic range, and the considerable morphological diversity. The primary objective of our research was to analyze the genus Dendrobium (focusing primarily on the nominal section) using DNA sequences and to integrate these findings with morphological data.

**Results:**

UPGMA morphological analysis and SIMPER analysis, considering 14 characters, showed that characters such as the placement of the inflorescence, the pseudobulbs from which the inflorescence grows, the surface of the lip, and the number of lobes on the lip are the distinguishing characters of the taxa within *Dendrobium *sensu lato.The nominal section, *Stachyobium*, and *Formosae* (species-rich) are more morphologically variable and exhibit a wide range of variability, in contrast to sections with fewer species. The reconstruction of the ancestral states of *Dendrobium *sensu lato showed that most of the characters examined arose independently several times during evolution. The only exception is dorsiventrally compressed leaves, a character that arose only once. In addition, the nominal section is not monophyletic and is not entirely consistent with morphology, and species of *Holochrysa*, *Breviflores* and *Stuposa* are closely related.

**Conclusion:**

We conclude that convergent evolution likely occurred among *Dendrobium *sensu lato representatives, and that many floral traits may have resulted from adaptations to pollinators. This has led to numerous classification problems within the genus. We have not received a clear answer to the question of how to classify species across the genus. Therefore, in this study, we focused primarily on the nominal section.

**Supplementary Information:**

The online version contains supplementary material available at 10.1186/s12870-025-06263-w.

## Introduction

With about 1,600 species and a wide geographical distribution *Dendrobium* Sw. is one of the largest genera in the Orchidaceae [1–6]. Species in this group are found in regions of southern, eastern, and southeastern Asia, the Pacific Islands, Australia, New Guinea, and New Zealand [7, 8]. Thus, this group represents one of the most complicated taxonomic challenges in the orchid family. In addition, there is a problem of correct identification due to the strong polymorphism observed among *Dendrobium* species [4, 9–11], lack of comprehensive taxonomic revisions and frequent description of species without verification of reference materials, which, *nota bene*, has been lost in many cases. Species in this group are mainly epiphytes with a sympodial type of growth. However, lithophytes and terrestrial plants are quite frequent [7, 8]. Pseudobulbs are highly diversified. There are both green and succulent forms, as well as thin, long, and cane-like ones [8, 12], and any intermediate types. Leaves are conduplicate and arranged in two rows. They can be dorsiventrally flattened, bilaterally flattened, or terete as well. Flowers are generally small to medium-sized, occurring either singly or more commonly in clusters with inflorescences placed apically or subapically on the pseudobulbs. Most often (but not exclusively), they come in colors such as white, purple, or yellow or a combination thereof. They vary in size, shape, persistence, and growth rate [9]. The lip may be 3-lobed, with a characteristic claw and striations or ridges present [8, 9]. In addition, there are other structures existed in some representatives, such as trichomes and papillae, which most probably are related to pollination [13, 14]. The gynostemium or column is cylindrical-conical. The column foot is typically very long and substantial [12, 15] Together with the lateral sepals and lip claw, they can be joined in various ways to form the spur-like mentum, a characteristic structure of this group [9]. The rostellum is swollen, and when touched, it releases an opaque liquid that attaches the pollen to the pollinator [9, 15]. Also present are 4 naked, laterally compressed pollinia, arranged within the anther in two pairs side by side [15]. The fruit is a dry, hairy capsule in which small seeds containing a spherical or ovoid embryo are usually present [8, 9, 16].

Unfortunately, few studies have been done on pollination, a phenomenon playing a key role in the evolution and continuity of the species. It is likely that some species, such as *Dendrobium unicum* Seidenf. from the nominal section, are pollinated by bees or wasps carrying pollen on their backs [9, 14]. Representatives of the sections *Dendrocoryne* Lindl. & Paxton, *Rhizobium* Lindl. & Paxton, and *Monophyllaea* Benth. mainly use pollinators from the genus *Trigona* Jurine, but also *Homalictus* Cockerell, *Lassioglossum* Curtis, or *Hylaeus* Fabricius [9, 17]. *Dendrobium setifolium* Ridl., which belongs to the section *Crumenata* Pfitzer, is also pollinated by the stingless bee of the genus *Trigona* [18]. *Amegilla* Friese probably can also pollinate species of this genus, mainly from the sections *Spatulata* Lindl. and *Calcarifera* J.J. Sm*.*, while *Xylocopa* Latreille from the section *Latouria* Miq. [9]. Birds can also serve in pollination by species belonging to the sections *Pedilonum* Blume, *Oxyglossum* Schltr. and *Calyptrochilus* Schltr.. In this case, pollen adheres to their beak [9, 19]. It is known that most representatives of this genus do not produce nectar, so pollinators are often deceived. In addition to bees and birds, other animals can pollinate *Dendrobium*. *Bombus* Latreille probably pollinates species in the sections *Formosae* (Benth. & Hk.f.) Hk.f. and *Epigeneium* Gagnepain [9]. A study by Kjellsson and co-authors confirms these data. In this research, *Bombus eximus* Smith was observed pollinating *Dendrobium infundibulum* Lindl [20]. Self-pollination was also reported, but rarely, for example in *Dendrobium striolatum* Rchb.f (section *Rhizobium*). It is most often caused by a lack of suitable pollinators [9].

## Background and goals

The history of *Dendrobium* is quite complicated and complex. In 1799, Swartz described this genus based on the presence of 5 spreading and upright petals with lateral sepals attached to the lip forming a "horn" [21]. The last characteristic was only mentioned, but it was not clearly defined. It was not until 1810 that Brown defined and named it as the mentum and a key feature of this genus. Later researchers also treated this character as taxonomically important [9]. It is worth noting that mentum occurs in several other groups of orchids, such as the Asian Vandeae and the Neotropical Maxillariinae, so it is not a feature unique only to *Dendrobium*. Since the description of the genus, different concepts have been proposed regarding its taxonomy and classification, which have been based on both morphological and molecular analyses.

Initially, Lindley (1830) placed *Dendrobium* with 19 other genera in Dendrobiinae G. Don. In *Dendrobium*, however, he distinguished four sections [22]. A few years later, the number of sections was increased to 10 [23]. This taxonomic concept focused primarily on floral characters, especially pollen, and less on vegetative traits. Whereas Schlechter divided *Dendrobium* into 4 subgenera and 41 sections based on the presence or absence of a sheath leaf base [24]. This division was considered completely artificial by Schuiteman [25]. However, some researchers, after making several modifications, adopted Schlechter’s system. For example, Brieger (1981) classified 28 genera in Dendrobiinae, most of which correspond to Schlechter's sections [4]. The other division was presented by Dressler (1993) and Wood (2006). They distinguished 6 and 5 genera, respectively, in the subtribe [9, 19]. At the end of the twentieth century, most authors distinguished the following genera in Dendrobiinae: *Dendrobium*, *Diplocaulobium*, *Cadetia*, *Flickingeria*, *Epigeneium*, and *Pseuderia* [26].During this period, the first results of molecular analyses of representatives of the Dendrobiinae have also been presented, primarily based on plastid markers sequences. Taxa of this subtribe were grouped on the three major clades. The first one included *Dendrobium* species found in continental Asia and western Malaysia (including the generitype *D*. *moniliforme* (L.) Sw.). The second one included *Dendrobium* along with the following genera: *Cadetia*, *Diplocaulobium*, and *Flickingeria*, found mainly in Australasia and the Pacific Islands. While the last clade included species of *Epigeneium* [2, 26–30]. Subsequent molecular studies based on additional nuclear markers correlated with the initial results. Tsai et al. presented the phylogeny of *Dendrobium* species from Taiwan [31]. In 2008, Burke et al. performed molecular analyses on the Australian section *Dendrocoryne* [32]. However, as in the aforementioned study, molecular analyses do not always agree with species morphology, and often the sections analyzed are still not monophyletic. Also worth mentioning are the works of Clements and Jones (2002) and Clements (2003), in which *Dendrobium sensu l*ato was divided into 3 subtribes: Dendrobinae, Grastidiinae and Epigeneiinae with about 50 genera to obtain monophyly. Their results are based on analyses using the ITS marker [33, 34]. In 2011, Schuiteman and Adams showed a different concept for classifying the representatives of *Dendrobium *sensu lato. They proposed to include several genera: *Cadetia, Diplocaulobium, Epigeneium, Euphlebium, Flickingeria* and *Grastidium* in *Dendrobium *sensu* latissimo* [25]. In 2013, Xiang et al. conducted analyses of the genus by interpreting various molecular markers and based on extensive sampling. The results of the phylogenetic analyses indicate two major clades in the studied group. In these clades, smaller subclades are distinguished, with sections that often exhibit a polyphyletic and paraphyletic character [4]. The taxonomy of this genus is more complex than previously thought [4, 9, 30]. In the present study, we have focused our attention on the nominal section of *Dendrobium*, which, of course, includes the type species of the entire genus. This section comprises species that exhibit clear morphological similarities to the generitype. The boundaries between sections are subjective and depend on the author's interpretation. In our view, individual sections should demonstrate some degree of morphological discontinuity. Therefore, it is crucial to define the scope of the nominal section, both in terms of its morphological characteristics and phylogenetic content. This paper prioritizes and emphasizes this aspect.

Despite numerous attempts to establish a clear classification, many uncertainties persist, and the taxonomy of the group remains unresolved. The aim of our study is to analyze the genus *Dendrobium* using all available DNA sequences and to integrate the results with morphological data. From our perspective, floral traits are of paramount importance in conducting such studies. Therefore, we focused primarily, though not exclusively, on these traits. The choice of appropriate markers is also crucial. Based on the findings of other researchers [5, 35, 36], the most reliable marker is nuclear ITS, which is therefore primarily used in this study to discuss phylogenetic relationships.

## Materials and methods

### Materials

DNA sequences of *Dendrobium *sensu lato taxa and *Bulbophyllum* species (used as the outgroup) were downloaded from the NCBI database (https://www.ncbi.nlm.nih.gov/genbank/). First, we used all the sequences available in this database and performed preliminary analyses. Because of the need to minimize the resulting tree, we selected taxa that represented particular sections and then used them to generate the final trees. For some species, we obtained sequences from plant material presented in Burzacka-Hinz et al. (2022) [13]. The GenBank accession numbers of the sequences used are given in Additional file 1 (Table S1). The nominal section has been treated as sensu lato, with all the species that have ever been part of it.

### Molecular analyses

We analyzed most of the markers available in GenBank (nuclear ITS and three plastid markers: *rbc*L, *mat*K and *trn*L-*trn*F). In addition, we performed an analysis for the low copy gene *Xdh*. First, the sequences for each marker were aligned using MAFFT v. 7 [37]. The minor errors were corrected in SeaView v. 5.0 [38]. On the *trn*L-*trn*F matrix, we observed highly variable and ambiguously aligned characters. These were excluded from the analysis. In accordance with Givnish et al. (2015), a selection of *Bulbophyllum* species were used as an outgroup [39]. Substitution models were calculated using the following website http://www.atgc-montpellier.fr and based on the AIC criterion, GTR + G + I was selected for the ITS and plastid matrices, and HKY + G + I for the *Xdh* dataset. Then, to eliminate possible incompatibilities in the topologies, we performed the Bayesian inference (BI) for each individual dataset: nrITS (820 bp), *Xdh* (756 bp), *mat*K (1624 bp), *rbc*L (1229 bp), *trnL-trn*F (1065 bp). On the CIPRES Science Gateway [40], we used MrBayes 3.2.7a with Markov Chain Monte Carlo (MCMC). Two simultaneous runs of four chains for 3 000 000 generations, sampling one of every 100 trees, were performed until the average standard deviation of the split ranges reached a value below 0.01. The first 25% of the trees were deleted as a burn-in, and the rest of the saved trees were collated into a majority consensus tree.

The plastid marker matrices proved to be uninformative and the resulting trees were polytomous. These are available in a corresponding author. Despite the fact that the topology of the trees obtained for the nuclear datasets is similar, for further analyses we have chosen the tree of the ITS matrix. The main reason was the larger number of samples. In addition, the *Xdh* tree was presented in Burzacka-Hinz et al. (2024) [41]. For the ITS dataset, we also performed maximum likelihood analysis (ML) using raxmlGUI 2.0 [42]. 1000 bootstrap replicates were used. The topology of the two trees, the BI analysis and the ML analysis were similar. Therefore, we edited the majority consensus tree with FigTree v.1.4.4 (http://tree.bio.ed.ac.uk/software/figtree/) and InkScape, but the support on the nodes was presented by both posterior probability (PP) and bootstrap (BS). We considered values of 0.95 or higher for PP and higher than 75 for BS to be strongly supported [43, 44].

### Ancestral state reconstruction (ASR) of morphological features

The ancestral states of morphological characters for *Dendrobium* species were reconstructed for 14 morphological features, using Mesquite v. 3.70 and the parsimony ancestral state reconstruction method [45]. The phylogenetic tree, obtained from Bayesian analysis was used as an input for the analysis. Numbers above the branches indicate the PP/BS support values. Taxa characters were coded for the presence (1,2 – yes) or absence (0 – no) of a feature. All character states are presented in Table S2 (Additional file 2).

### Analysis of morphological variation

To summarize the morphological variation in *Dendrobium *sensu lato, as well as between species belonging to 39 sections, we described 318 taxa using 14 well-distinguished characters (Table S3 and S4 in Additional file 3). The selected traits referred to apparent external differences for pseudobulbs, leaves and inflorescences (Ch1-9), as well as floral characters (Ch10-14). To examine morphological variation, the listed traits were binary coded < 0,1 > , and were often of opposite natures, with the exception of the trait describing leaf cross-section—Ch5, which was included in three states of this trait < 0,1,2 > (Table S3 in Additional file 3). Similarity between taxa were calculated using the Jaccard similarity coefficient. Based on this data set (Table S4 in Additional file 3), a cluster analysis was performed, and then a dendrogram was constructed using the UPGMA method. In addition, to describe the extent of morphological variation among *Dendrobium* sections and the interrelationships between them, we conducted a principal coordinate analysis (PCoA) and a non-metric multidimensional scaling analysis (NMDS), based on the Jaccard similarity matrix in both cases. To identify the morphological characters that contributed most to the observed differences between *Dendrobium* taxa, we carried out a SIMPER (SIMilarity PERcentage) analysis, which attempts to assess the average percent dissimilarity of each variable between specimens in the Bray–Curtis similarity matrix. The average percentage changes of each *Dendrobium* section in the differences in the Bray–Curtis similarity matrix are also summarized in Table S5 (Additional file 3).

Additional analyses but based on only 5 floral traits (Ch10-Ch14), were conducted in the same manner as described above. The difference was that the cluster analysis was performed using Ward's method, which allows clusters to be combined in such a way as to minimize the increase in within-group variance. This method is very effective, although it aims to create clusters of small size, but represents the most essential clustering of individuals [46]. All multivariate analyses and tests were performed using software packages: STATISTICA v. 13 and PAST v. 4.14 [47, 48].

## Results

### Phylogenetic analysis

**Fig. 1:**
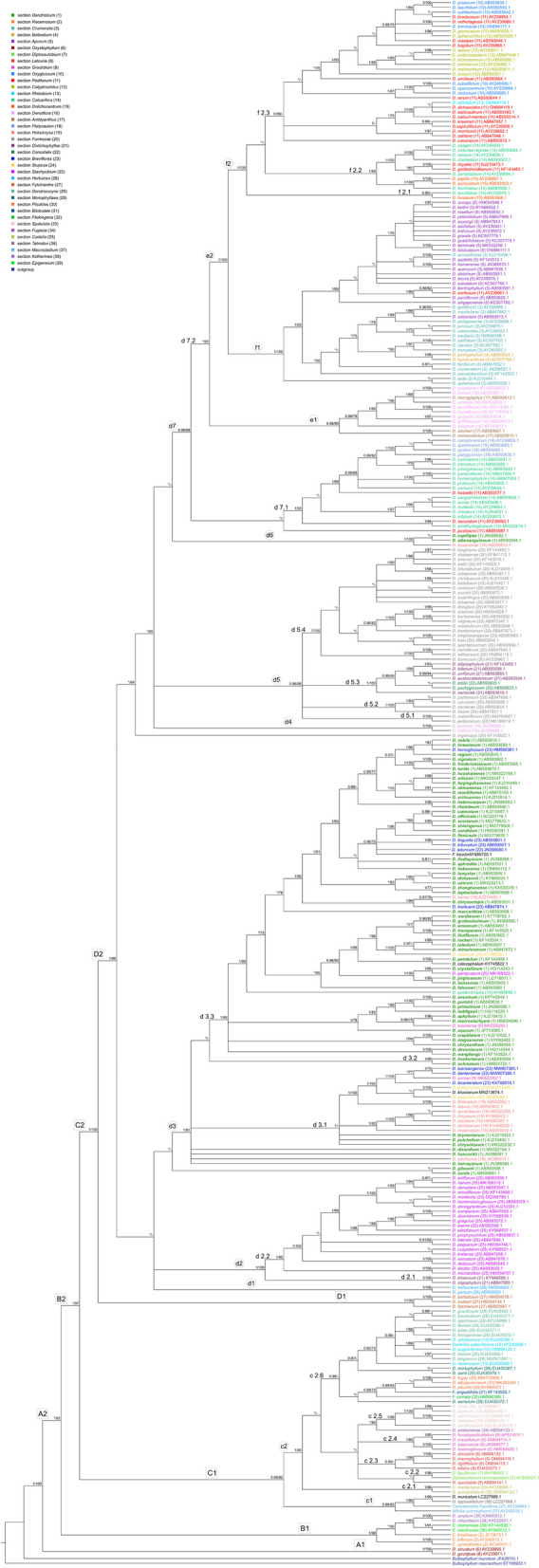
The first part of the 50% majority rule consensus tree for *Dendrobium *sensu lato, obtained for the nrITS dataset using Bayesian inference. Numbers above branches indicate posterior probability and bootstrap support values from maximum likelihood analysis (PP/BS); PP values < 0.95 are marked with an asterisk, and BS values < 75% are marked with a -. Letters with numbers above branches mark clades discussed in the text. Numbers in parentheses next to taxon names indicate section affiliation. In addition, each section is highlighted in a different color and the species of the nominal section are bolded

*Dendrobium *sensu lato is a monophyletic group that evolved from a common ancestor, according to the molecular analysis of the nuclear marker ITS (Fig. 1). Species such as *Epigeneium*, *Diplocaulobium* or *Flickingeria* occupy basal branches alongside *Dendrobium *sensu stricto*,* without forming a separate clade. Clade **A1** (Fig. 1) includes species of the section *Oxystophyllum*, while clade **A2** (Fig. 1) comprises the remaining *Dendrobium* species with moderate support (BS = 82). Two main lineages, **B1** (PP = 1, BS = 98) and **B2** (PP = 0.92, BS = 97), diverge from clade **A2** (Fig. 1). **B1** includes taxa such as *Racemosum* and *Katherinea*, usually treated as separate sections or genera. Clade **B2** also splits into two lineages, **C1** (PP = 0.99, BS = 82) and **C2** (PP = 1, BS = 100) (Fig. 1). **C1** contains species belonging to different sections, including two subclades: **c1** (*D*. *oppositifolium* and *D*. *muricatum*) (Fig. 1) and the larger but poorly resolved **c2** with six polytomous lineages (**c2.1**-**c2.6**, Fig. 1). Species *D*. *spectabile* and *D*. *amboinense* remain ungrouped but are closely related, having evolved from a common ancestor (node **c2**). Further relationships within **c2** lack sufficient support.

Clade **C2** (Fig. 1) is divided into two groups. The basal group (clade **D1**, PP = 1, BS = 100) includes section *Fytchianthe*, while the second group (clade **D2**, Fig. 1) splits into two poorly supported lineages, forming seven polytomous subclades: **d1**, **d2**, **d3** (Fig. 1) and **d4**, **d5**, **d6**, **d7** (Fig. 1). Each subclade may represent an independent evolutionary lineage. Subclade **d1** (PP = 1, BS = 100) includes two species from section *Herbacea*, while **d2** (PP = 1, BS = 100) comprises two groups: **d2.1** *(Distichophyllae*, PP = 1, BS = 100) and **d2.2** (*Stachyobium*, PP = 1, BS = 95). Subclade **d3** (Fig. 1, PP = 1, BS = 91), the largest but non-monophyletic group, includes taxa from sections *Holochrysa*, *Breviflores*, *Stuposa*, and nominal, along with *D*. *braianense* (sect. *Holochrysa*), *D*. *albosanguineum*, and *D*. *capillipes* (sect. *Dendrobium*). Subclade **d3** also contains species previously classified in other sections (e.g., *Stachyobium*, *Grastidium*) but exhibits numerous unresolved polytomies. Subclade **d4** (Fig. 1, PP = 1, BS = 100) contains two species from section *Densiflora*. Subclade **d5** (PP = 0.96, BS = 86) includes four polytomous lineages: **d5.1**, **d5.2**, **d5.3**, and **d5.4** (Fig. 1, PP = 1, BS = 100). Groups **d5.1**, **d5.2**, and **d5.4** represent section *Formose*, while **d5.3** includes taxa from sections *Conostalix* and *Distichophyllae*. Subclade **d6** (Fig. 1, PP = 1, BS = 100) groups species from sections *Holochrysa* and *Dendrobium*.Subclade **d7** (Fig. 1, PP = 0.98, BS = 89) splits into two groups. One, **d7.1** (Fig. 1, PP = 1, BS = 93), includes species from sections *Calcarifera*, *Platycaulon*, and two species of *Pedilonum*. Notably, some *Calcarifera* species also appear in subclades **f2.1** and **f2.2** (Fig. 1) alongside species from *Dolichocentrum* and *Pedilonum*. Subclade **d7.2** (Fig. 1) comprises two subclades, **e1** (Fig. 1, PP = 0.98, BS = 89) and **e2** (Fig. 1, PP = 1, BS = 100), both with strong bootstrap support (BS = 93). Subclade **e1** includes species from *Densiflora*, except *D. jenkinsii* and *D. lindleyi*, which form subclade **d4** (Fig. 1). It also contains *D. melanosticum*, *D. stocari*, and *D. microglaphys* from *Amblyanthus*. Subclade **e2** divides into groups **f1** and **f2** (Fig. 1). Group **f1** (PP = 1, BS = 100) includes species from *Crumenata* and *Aporum*, with exceptions: *D. pachyphyllum* and *D. hymenanthum* (*Bolbidium*), and *D. confusum* (*Pedilonum*). Group **f2** (PP = 1, BS = 100) likely evolved into three polytomic groups: **f2.1** (PP = 0.95), containing *D. furcatum* (*Dolichocentrum*) and *D. lancifolium* (*Calcarifera*); **f2.2** (PP = 1, BS = 99), comprising species from *Calcarifera*, *Dolichocentrum*, and *Pedilonum*; and **f2.3** (PP = 0.99), with taxa from *Calyptrochilus*, *Pedilonum*, *Rhizobium*, and *Oxyglossum*.

### Ancestral state reconstruction (ASR) of morphological features

The reconstruction of the ancestral states of *Dendrobium* features is presented in 4 different phylogenetic trees (Figs S1-S4, Additional file 2). States for characters: 1. Pseudobulbs separated (green color)/approximate; 2. Pseudobulbs and leaves hairy (red color)/glabrous; 3. Pseudobulbs leafy at the apex (light blue color)/leafy througout; 4. Pseudobulbs swollen (dark blue color)/reedlike stem present in the Fig. S1 (Additional file 2). The reconstruction of following features are given at the Fig. S2 (Additional file 2): 5. Leaves laterally (green color)/dorsiventraly (red color) compressed; terete or subterete (dark blue color); 6. Leaves thick, succulent (light blue color) are reconstructed at Fig. S2 (Additional file 2). Fig. S3 (Additional file 2) presents the reconstruction for characters: 7. Inflorescence elongate (green color)/short; 8. Inflorescence produced on last year’ (red color)/current pseudobulbs; 9. Inflorescence placed laterally (dark blue color)/apically on/along pseudobulbs. Finally, the reconstruction of: 10. Lip 3-lobed (red color)/unlobed, 11. Lip surface tomentose, papillose, glandular to hairy (green color)/glabrous, papillate or wrinkled; 12. Lip callus absent (dark blue color)/prominent; 13. Mentum as long as or longer (light blue color)/shorter than dorsal sepal is shown in Fig. S4 (Additional file 2).The results of the analysis suggest that the last common ancestor of *Dendrobium *sensu* lattisimo* was characterized by approximate, glabrous, leafy (throughout) and reed-like pseudobulbs, dorsiventrally compressed leaves, inflorescence placed apically on current pseudobulbs and glabrous, papillate or wrinkled, unlobed lip with prominent callus and mentum shorter than dorsal sepal. In addition, most of the characters examined arose independently several times during evolution and were then lost in different lineages. The only opposite to this is dorsiventrally compressed leaves, a character that arose only once, but has also been lost in species of different *Dendrobium* sections.

### Morphological similarities

**Fig. 2:**
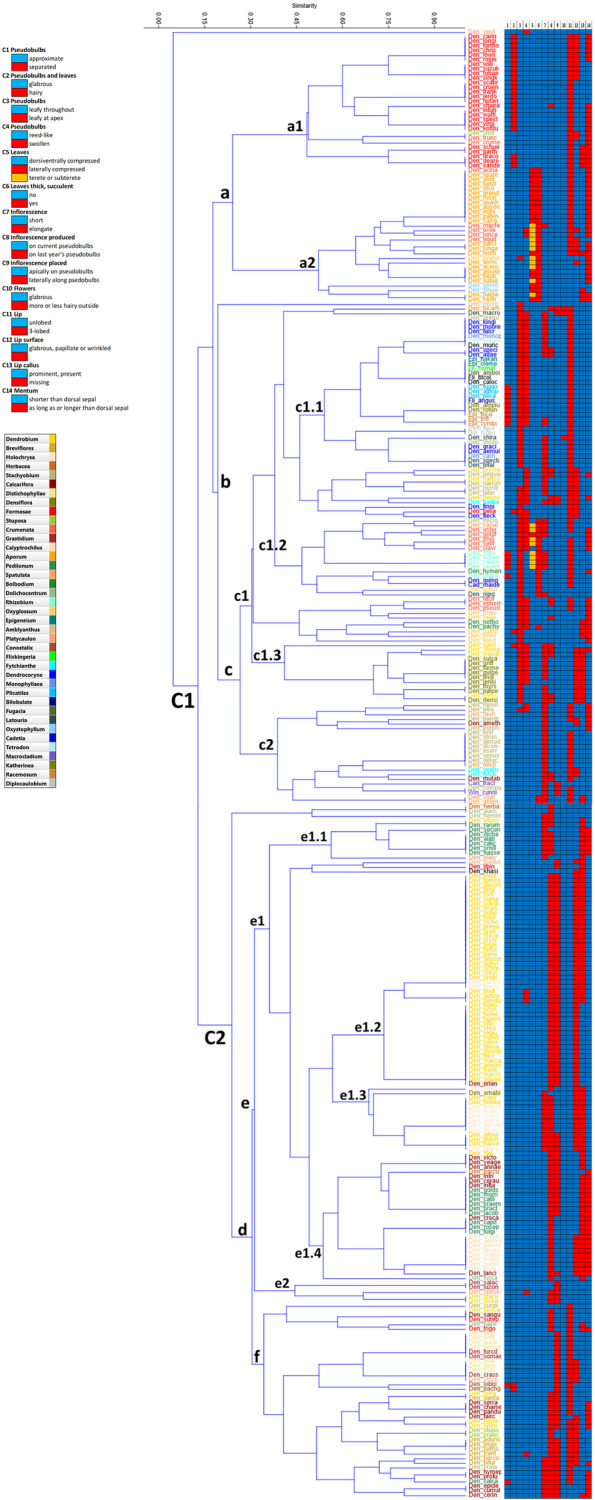
UPGMA analysis based on Jaccard's similarity coefficient, showing morphological similarity among specimens of *Dendrobium *sensu lato. The clusters and subclusters that are the subject of this discussion are indicated by upper and lower case letters and numbers. A detailed description of the morphological characters can be found in Table S3 in Additional file 3

To describe mutual resemblance in *Dendrobium *sensu lato, a similarity matrix for the 14 morphological characters studied was used in a hierarchical cluster analysis. The morphological clustering cophenetic coefficient with the Jaccard similarity matrix was 0.786. The resulting UPGMA dendrogram separated specimens into two main clusters, where *D. ypsilon* remained distinct in the upper part of the dendrogram (Fig. S5 in Additional file 3). The two clusters differed primarily in traits describing: the appearance of inflorescences on current vs. last year's psedudobulbs (Ch8); the position of inflorescences on psedudobulbs (apically vs. laterally) (Ch9); lip shape (unlobed vs. three-lobed) (Ch11); lip surface (Ch12); and the presence of lip callus (Ch13) (Table S3 in Additional file 3). The first cluster (**C1**) included specimens from *D. cariniferum* to *D. antennatum* and corresponded to species in which the inflorescences are primarily produced on the current pseudobulbs and are apically placed on them, the lips are mostly divided into 3-lobed structure, with glabrous, papillate or wrinkled surface, and with prominent and present callus. In contrast, the second cluster (**C2**) included the remaining species from *D. herbaceum* to *D. cerinum* and the species grouped here have inflorescences mostly laterally placed on last year’s pseudobulbs, the lips are unlobed, with a usually tomentose, papillose, glandular or hairy surface, and no callus in most species in this group. Cluster **C1** revealed three smaller subclusters (**a, b** and **c**) (Fig. 2). Subcluster **a** contained species from *D. cariniferum* to *D. keithii* and was further divided into two subgroups (**a1** and **a2**). The first of these, **a1** (from *D. cariniferum* to *D. sanderae*) included species characterized by hairy pseudobulbs and leaves (Ch2), where the leaves are dorsiventrally compressed and thin (Ch5 and Ch6). Representatives of section *Formosae* have been grouped here. In contrast, the second subgroup, **a2** (from *D. acinaciforme* to *D. keithii*) included species characterized by glabrous pseudobulbs and leaves (Ch2), where the leaves are laterally compressed, terete or subterete and thick (Ch5 and Ch6). Representatives of the sections *Aporum* and *Oxystophyllum* belong here. Distinguished below is subcluster **b**, which is further subdivided into the separate, single species *D. microglaphys*, and the extensive and highly diverse subcluster **c**, from *D. bicameratum* to *D. antennatum*. This subcluster was divided into two groups (**c1** and **c2**), and within the **c1** group, three subgroups were distinguished (**c1.1**, **c1.2**, **c1.3**). Group **c1** (from *D. bicameratum* to *D. densiflorum*) from group **c2** (from *D. minutiflorum* to *D. antennatum*) was separated on the basis of Ch3 and Ch4 characters, where species in group **c2** have pseudobulbs with full-length foliage, and the leaves are reed-like. This group included species from sections *Spatulata* and *Fytchianthe*. A smaller subgroup **c1.1** (from *D. bicameratum* to *D. bifalce*) was consistent with species with pseudobulbs leafy at the apices and swollen (Ch3 and Ch4), where the lips are 3-lobed with present and prominent callus (Ch11 and Ch13); another subgroup **c1.2** (from *D. brymerianum* to *D. laevifolium*) has quite often thick leaves (Ch6); and subgroup **c1.3** (from *D. senile* to *D. densiflorum*) was determined by the traits associated with unlobed lip and lack of callus (Ch11 and Ch13). Species belonging to the sections *Latouria*, *Dendrocoryne*, *Monophyllaea*, *Epigeneium*, *Plicatiles*, *Katherinea*, *Racemosum* and *Diplocaulobium* were represented in subgroup **c1.1**. Subsequently, specimens from the sections *Rhizobium*, *Cadetia*, *Bolbodium* and *Oxyglossum* were grouped in subgroup **c1.2**, while subgroup **c1.3** was designated by section *Densiflora*. The second cluster (**C2**) appears to be more homogeneous and less diverse, and the species belonging here are characterized by inflorescences placed laterally along pseudobulbs that are of last year (Ch8 and Ch9), and the lip surface is tomentose, papillose, glandular to hairy (Ch12), with a few exceptions (Fig. 2). This cluster was divided into two subclusters—**d**, extensive but uniform, and a smaller one with two species, *D. herbaceum* and *D. auriculatum*, at the top. Subcluster **d** was further divided into two groups—**e** and **f**, differing primarily in the Ch11 trait. Group** f** includes species having a 3-lobed lip for this trait and consists of specimens from *D. cuspidatum* to *D. cerinum*, mainly from the sections *Distichophyllae*, *Conostalix* and *Breviflores*. In turn, group **e** was subdivided into two subgroups (**e1** and **e2**), while the **e1** subgroup was split into four smaller subgroups (**e1.1**, **e1.2**, **e1.3**, **e1.4**). To subgroup **e1** belongs species from *D. hemimelanoglossum* to *D. furcatum*, while subgroup **e2** aggregates species from *D. salaccense* to *D. dickasonii*. Identification of subgroup **e1.1** (from *D. hemimelanoglossum* to *D. platygastrium*) was based on features associated with an elongated inflorescence (Ch7) and a glabrous, papillate or wrinkled lip surface (Ch12). Some species of sect. *Pedilonum* belong here. Another subgroup **e1.2** (from *D. melanostictum* to *D. prianganense*) was distinguished on the basis of the short inflorescence (Ch7), which is placed laterally along pseudobulbs (Ch9) and the different lip surface (Ch12) than in the case of **e1.1**. Most species belonging to this subgroup are assigned to the broad *Dendrobium* section. In the smaller subgroup **e1.3** (from *D. lohohense* to *D. lituiflorum*), species have an elongated inflorescence (Ch7) which in turn is in most located apically on pseudobulbs (Ch9), and lip callus is absent (Ch13). Mainly representatives from section *Holochrysa* are characterized by such morphology. The last subgroup—**e1.4** within the **e1** group, included species from *D. victoriae-reginae* to *D. furcatum*, which have a short inflorescence (Ch7), a glabrous, papillate or wrinkled lip surface (Ch12), and above all, this subgroup is distinguished by having a mentum as long or longer than the dorsal sepal (Ch14).

Additional cluster analysis was carried out to describe patterns of variation in *Dendrobium *sensu lato but based on 5 floral characters (Ch10-Ch14; Table S3 in Additional file 3). In this case, the cophenetic correlation coefficient was 0.774, and this is a good value for how accurately the resulting dendrogram reflects the pairwise distances between the original unmodeled data. Dendrogram obtained using Ward’s method divided the specimens into two main clusters (**Cf1** and **Cf2**), in which six predominant patterns of floral variation can be observed (Fig. S6 in Additional file 3). The first floral cluster (**Cf1**) included species from *D. densiflorum* to *D. yeageri*, corresponding to species in which the flowers are glabrous with unlobed lips, where the surface is mostly tomentose or papillose, glandular to hairy; the callus is absent, and the mentum is shorter than the dorsal sepal. All species of the broad section *Dendrobium* are grouped here. In turn, the second floral group (**Cf2**) is more diverse and includes species from *D. shiraishii* to *D. fulgidum*, and the species grouped here also have glabrous flowers, but the lips are trilobed. However, in terms of the other three traits, this group is more variable, and it is the type of lip surface, the presence or absence of callus, and the length of the mentum in relation to the dorsal sepal that distinguish the smaller subgroups. The exceptions are three species in the upper part of this cluster (*D. shiraishii*, *D. bicameratum*, *D. macrophyllum*), which have flowers that are more or less hairy on the outside, unlike the other representatives of this group.

In general, small sections are consistent in the two analyses with respect to morphology and flower patterns. On the other hand, large sections such as *Dendrobium*, *Stachyobium* or *Formosae* are already more morphologically variable and here the range of variation is already wider and varies depending on the set of characters analyzed.

**Fig. 3:**
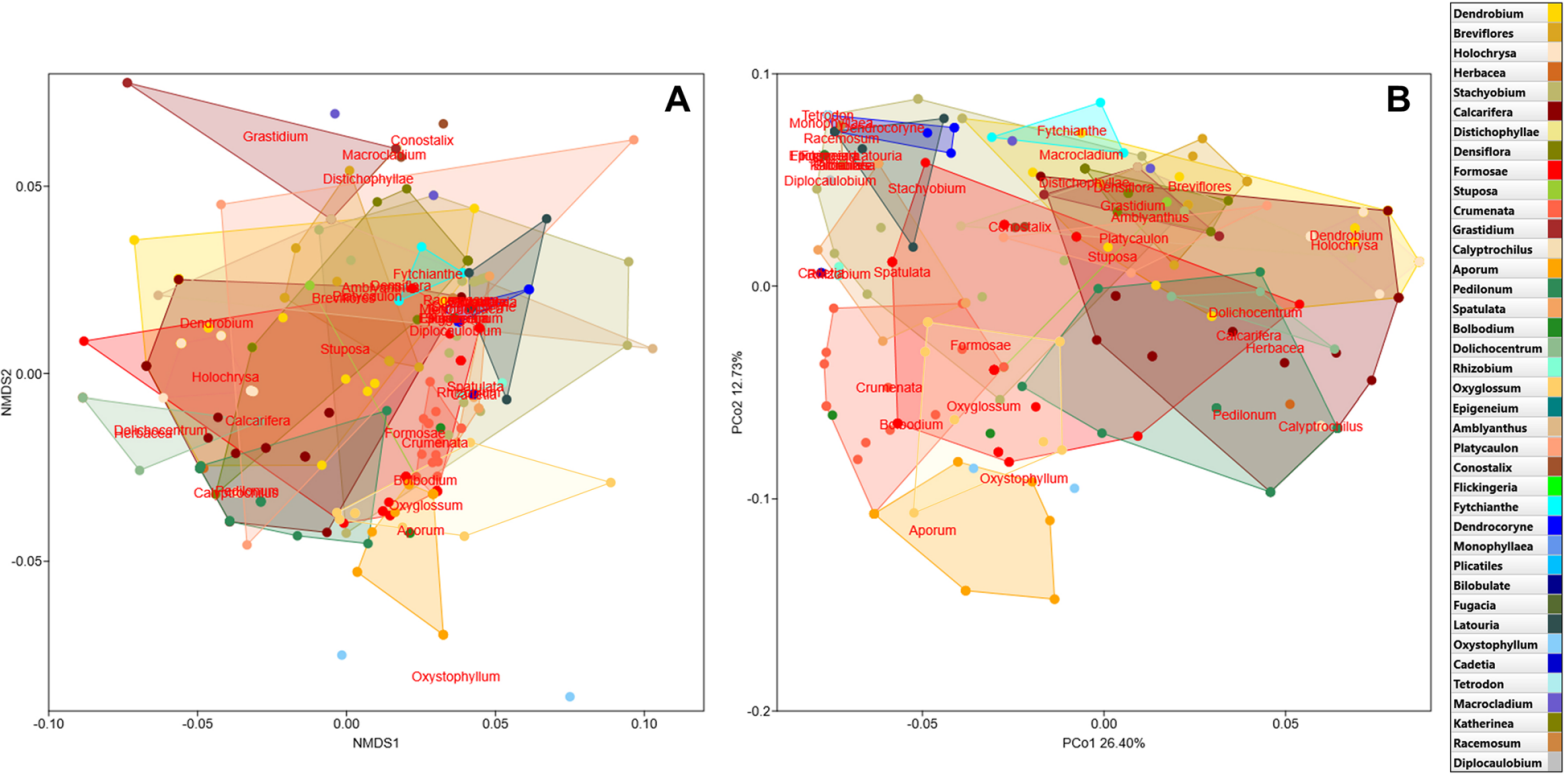
Non-metric multidimensional scaling analysis, NMDS (**A**) and principal coordinate analysis, PCoA (**B**) showing the two-dimensional ordering of *Dendrobium *sensu lato specimens, based on 14 morphological characters. Convex hulls for each *Dendrobium* section were added to the plots

SIMPER analysis identified morphological characters as the most differentiating ones, and these were the same traits described above that divided the *Dendrobium *sensu lato into two major, large clusters—C1 and C2. These were characters related to the position of inflorescences on pseudobulbs from the current or previous year (Ch8 and Ch9), and the presence or absence of a three-lobed lip (Ch11), as well as the type of lip surface (Ch12). The overall average dissimilarity was 69.18% for the morphological characters used comprehensively in our study (Table 1A). In contrast, in the analysis based on floral traits, the presence or absence of a three-lobed lip (Ch11) was identified as the trait that most differentiated *Dendrobium *sensu lato, and it was this trait that divided *Dendrobium* into two main groups (clusters Cf1 and Cf2) described above. The next three traits related to the lip surface, mentum length and callus occurrence (Ch12, Ch14 and Ch13, respectively) had similar average similarity values, while the trait related to flower hairiness (Ch10) was of negligible importance in the analysis. In this case, the overall average dissimilarity equalled 63.53% (Table 1B). NMDS and PCoA analyses showed a wide range of variability in *Dendrobium *sensu lato, but the sections overlapped considerably (Fig. 3). Despite the apparent and continuous morphological variation, the resulting image is the same as that visualized in the UPGMA cluster analysis. However, the cumulative percentage of explained variance by the first two axes in the PCoA analysis was only 39%, while the stress value was S = 0.474, indicating the low quality of the fit in NMDS. This is largely due to the number of minor variables used in the ordering, as well as the close morphological similarity of the taxa, where their ranges of morphological variation overlapped. In both cases, the division of sections among *Dendrobium *sensu lato was along the second axis. In the case of the NMDS, the left side of the scatter plot grouped representatives for sections associated with cluster C2 in the UPGMA (e.g., sections *Dendrobium*, *Calcarifera*, *Calyptrochilus*, *Grastidium* and *Pedilonum*) (Fig. 3A), while on the opposite side were species belonging to cluster C1. An almost identical picture (although it appears slightly more ordered) was obtained in the PCoA analysis, with the difference that on the left side of the plot were concentrated representatives of sections related to cluster C1, while on the other side of the axis were characteristic sections for cluster C2 (e.g., sections *Formosae*, *Aporum*, *Crumenata*, *Dendrocoryne*, *Oxyglossum*, *Spatulata*, *Rhizobium*, *Racemosum*) (Fig. 3B).

**Table 1:** SIMilarity PERcentage (SIMPER) analysis for 14 morphological characters (**A**) and only for 5 floral traits (**B**) showing the contribution of each trait in distinguishing between taxa within *Dendrobium *sensu lato. Overall average divergence was 69.18% for A and 63.53% for B, respectively. For a detailed description of the morphological characters, see Table S3 in Additional file 3 (Av. dissim.—average dissimilarity; Contrib. %—percentage of similarity explained by individual traits; Cumulat. %—cumulative percentage of Bray–Curtis similarity)

A) Morphological traits	Av. dissim	Contrib. %	Cumulat. %
8	7.43	10.75	10.75
11	7.41	10.70	21.45
12	6.96	10.06	31.51
9	6.77	9.78	41.29
14	6.55	9.47	50.76
13	6.36	9.19	59.95
7	6.17	8.92	68.87
3	5.52	7.98	76.85
4	4.44	6.41	83.26
5	4.01	5.80	89.06
6	3.54	5.12	94.18
2	2.56	3.70	97.88
1	1.23	1.78	99.66
10	0.23	0.34	100
B) Floral traits	**Av. dissim**	**Contrib. %**	**Cumulat. %**
11	18.17	28.60	28.60
12	15.90	25.02	53.62
14	14.72	23.16	76.78
13	14.18	22.32	99.10
10	0.569	0.896	100

In subsequent NMDS and PCoA analyses conducted only for floral traits, there was again a large overlap in the ranges of morphological variation, and thus a high similarity between the observed floral patterns for among individual *Dendrobium* sections (Fig. S7 in Additional file 3). The cumulative percentage of explained variance by the first two axes in the PCoA analysis was almost 70%, while the stress value was S = 0.378, indicating the low quality of the matching in NMDS. As with the overall analysis, the picture obtained here is very similar to that visualized in the UPGMA cluster analysis.

## Discussion

*Dendrobium* is a large genus with a broad geographical distribution. Despite numerous studies, a consistent classification for the group as a whole and for its individual sections has yet to be established. Comprehensive data to determine even the sectional affiliation of individual species remains lacking. The identification of morphological traits helps in understanding the genetic relationships between particular species [49]. Unfortunately, in the case of *Dendrobium*, such studies are insufficient to draw even preliminary conclusions about these relationships. It is a complex and challenging group. A similar situation can also be observed in other groups of orchids, such as the closely related genus *Bulbophyllum* [50], *Encyclia* [51] or *Pleurothalis *sensu lato encountered before Luer's fundamental works [52]. *Dendrobium* has a high morphological diversity and overlapping differences. This is confirmed by the results of Adams, Niu et al. and our own (Fig. 3 and Fig. S7 in Additional file 3) [11, 29]. As can easily be seen, the phylogeny of *Dendrobium* also needs to be clarified (Fig. 1), and what is more, it is not entirely consistent with morphology (Fig. 2 and Fig. S6 in Additional file 3). The result obtained for the ITS marker (Fig. 1) shows that *Dendrobium *sensu* latissimo* is a monophyletic group, contrary to the work of Clements [34]. However, our result also proves that the nominal section, as traditionally understood, is not monophyletic (Fig. 1). To clarify this situation, we focused on and combined two main aspects—morphological variability and phylogenetic relationships. This allowed us to more thoroughly analyze species from the nominal section and from the remaining sections where we had doubts about taxonomic affiliation, based on comprehensive information. The species belonging to the section *Dendrobium*, according to different authors and our results, are listed in Table S6 (Additional file 4).

A comparison of morphological similarity analyses, ancestral state reconstructions for morphological traits, and phylogenetic analysis showed that vegetative traits are much less variable than the floral ones. We suggest that floral traits in *Dendrobium* representatives evolved several times independently (Figs. S3 and S4, Additional file 2). Therefore, taxa placed in the same section did not always form a consistent clade on the phylogenetic tree, such as species in the nominal section. Morphological similarity analyses also did not fully reflect previously proposed sectional divisions. Section *Oxystophyllum* is presented by only two species in our study but they are included in one clade (A1, Fig. 1). However, in the morphological analysis, they were placed in two separate clusters, cf1.2c and cf1.2b (Fig. S6, Additional file 3), simply because *D*. *sinuatum* does not have a callus on the lip. In contrast, clade B1 (Fig. 1) is consistent. It includes species of *Racemosum*, *Epigeneium* and *Katherinea*. This time, the proposed sectional division was reflected in both the phylogenetic results and the majority of morphological features (Figs. 1, 2 and Fig. S6 in Additional file 3). Considering our results and the works of Clements or Pridgeon et al., one has to think about the position of these taxa (especially *Oxystophyllum*) [10, 34, 53]. With their inclusion in the genus as a whole, *Dendrobium* will still be a monophyletic taxon. Treating these taxa as separate genera, they are sisters to *Dendrobium *sensu stricto.

Worth considering is the section *Holochrysa*. This is a paraphyletic group, and some species seem to fit phylogenetically and morphologically into the nominal section (subclade d3, Fig. 1). Of note is *D*. *braianense*, which is grouped with *D. capillipes* and *D. albosanguineum* (section *Dendrobium*) on the ITS tree (subclade d6, Fig. 1). But these taxa do not join other species of the nominal section. Also, analysis of morphological similarity showed that these species grouped together and with other nominal section taxa (Fig. 2 and Fig. S6 in Addtional file 3). Most species in the typical section have inflorescences produced on last year's pseudobulbs and placed laterally along the pseudobulbs, *D*. *braianense* also has these, but additionally has an elongated inflorescence. We propose to include *D*. *brainense* in the section *Dendrobium* based on the results obtained. The question that comes to mind is whether the remaining species of the *Holochrysa* section should be included in the nominal section, as the results of our analyses and Xiang et al. suggest [4]. It is worth considering. On the ITS tree, representatives of this section were grouped as an independent polytomous lineage (subclade d3.1, Fig. 1) that evolved from the common ancestor, the same as taxa of the nominal section (node d3, Fig. 1). However, in d3.1 were also placed *D*. *chrysotoxum*, *D*. *pulchellum* and *D*. *brymerianum* (sect. *Dendrobium*), *D*. *stuposum* and *D*. *praecinctum* (sect. *Stuposa*), *D. dantaniense* and *D*. *bicameratum* (sect. *Breviflores*), *D*. *somae* (sect. *Grastidium*) and *D*. *khasianum*. The analysis of floral characters also aggregates taxa of *Holochrysa* together with species of the section *Dendrobium*, but the differences, e.g. in the structure of the inflorescences, make treating all species as one section still a matter of debate (Fig. 1, Fig. S3 in Addtional file 2, Fig. S6 in Addtional file 3). Also noteworthy is *D*. *henryi* (subclade d3.3, Fig. 1), which is representative of *Holochrysa*. The results, both phylogenetic and morphological analyses, prove that this taxon should be included in the nominal section (Figs. 1 and 2, Fig. S6 in Addtional file 3). In addition, similar conclusions obtained by Yuan et al. and Xu et al. [54, 55]. The last species from the section *Holochrysa* that we would like to mention, *D*. *lohohense*, was placed as an independent polytomous branch from node d3 (Fig. 1), from a potential common ancestor, as well as other species of the nominal section. The morphological similarity analysis (Fig. 2, Fig. S6 in Addtional file 3) shows that it clusters together with representatives of the section *Dendrobium*, but the only feature that distinguishes this species is the inflorescence placed apically on pseudobulbs (Fig. 2, Fig S6 in Addtional file 3). Reconstruction of the ancestral state indicates the plesiomorphic nature of this character (Fig. S3 in Additional file 2). Interestingly, the absence of an elongate inflorescence placed laterally along the pseudobulbs and having only the above variant is not characteristic of either *Dendrobium* or *Holochrysa* (Fig. 2, Fig. S6 in Addtional file 3).The representatives of the section *Breviflores* were also grouped together with the species of the typical section (clade d3, Fig. 1). This needs to be discussed like the taxa in the section *Holochrysa*. Surprisingly, *D*. *hercoglossum* (sect. *Breviflores*) formed a strongly supported clade (PP = 1, BS = 96, Fig. 1) together with *D*. *nobile*, *D*. *linavianum* and *D*. *regium* (sect. *Dendrobium*). This is puzzling, because analyses of morphological characters clearly group this species with the other taxa belonging to the *Breviflores* (Fig. 2, Fig. S6 in Additional file 3). It has also been considered *Breviflores* instead [7, 9, 54, 56–58]. In addition, the presence of elongated inflorescences and the other results obtained also led us to leave this species in its current group (Fig. S3 in Additional file 2). A similar situation occurs with the remaining species in this section.

We also noted several species that have never been placed in nominal sections but are grouped in clade d3 (Fig. 1), e.g. *F. bicolor*, *D*. *khasianum*, or *D*. *calocephalum*. However, based on our results, we believe that although they are closely related to species in the section *Dendrobium*, their morphology is so different that they should be excluded from the typical section.

*D*. *capillipes* and *D*. *albosanguineum* (clade d6, Fig. 1) were placed beyond the clade with the section *Dendrobium* (clade d3, Fig. 1). UPGMA analysis for 14 morphological characters divides that both species are clustered with representatives of the nominal section (cluster C2, Fig. 2), but in different subgroups, e1.3b and e1.1. There are no differences in pseudobulbs or leaves, probably these characters are relatively consistent in all taxa of the typical section (Fig. 2). In the analysis of the morphology of the floral pattern, these two species are also divided into two subgroups (cf1.2c and cf1.1, Fig. S6 in Additional file 3). In this case the differences are more pronounced. Despite the presence of some deviations, it seems to us that both species should be included in the nominal section, as recognized by Peyachoknagul et al. [58]. However, we have more doubts in the case of *D*. *capillipes*. The number of leaves in this species varies from three to five. Takamiya et al. show that this is unique for both sections *Holochrysa* and *Dendrobium* [30]. Nevertheless, they treated this species as *Holochrysa*, while Wang et al. classified it in the section *Dendrobium*, and Schuiteman and Xiang et al. treat this taxon as unplaced [2, 4, 7]. *D*. *gibsonii* and *D*. *senile* were also not connected to the nominal section. They form independent branches that evolved from the ancestor at node D2 (Fig. 1). Takamiya et al. treated *D*. *gibsonii* as part of the section *Holochrysa* [30]. However, the results of our analyses do not confirm the close relationship with taxa of this section. Moreover, the correct assignment of taxa to these two sections is still controversial and the results so far are inconclusive. There are many morphological characters that suggest a combination of these sections. This is also supported by our molecular analysis. Although *D*. *gibsonii* is phylogenetically outside the d3 clade (Fig. 1), in our opinion, its set of characters allows this species to belong to the section *Dendrobium* (Fig. S6 in Additional file 3). This proposal has been confirmed by other authors [7, 56, 57]. The situation is different in the case of *D*. *senile*. Not only the phylogeny, but also the vegetative and floral characters do not correspond to the remaining species of the nominal section (Fig. 1 and Fig. S6 in Addtional file 3). Therefore, we propose that this species be separated from the section *Dendrobium*. Interestingly, Wood included this species in the section *Formosae* [9], and Xiang et al. treated it as an unplaced species [4].

We were also interested in two other species, *D*. *brymerianum* and *D*. *hancocki*, which Wood placed in the sections *Densiflora* and *Holochrysa*, respectively [9]. The morphological analyses carried out also do not indicate that they belong to the typical section (Fig. 2 and Fig. S6 in Additional file 3). It should be noted that on the phylogenetic tree obtained, they are included in the nominal section (clade d3, Fig. 1). Despite their phylogenetic affiliation, we prefer to separate these species from the nominal group. Although some authors put them here [35, 57]. In the case of *D. brymerianum*, apart from morphological differences, we noticed its relatively narrow and unusual range of occurrence compared to other species of the type of section. *D*. *zhenghuoense*, the species that was grouped with most taxa of the nominal section in the strongly supported clade d3 (PP = 1, BS = 98, Fig. 1), is morphologically distinct. The lack of callus on the lip, the mentum as long as or longer than the dorsal sepal, the elongate inflorescence, and the presence of swollen and leafy pseudobulbs at the apex are convergent characters that appear several times, probably in response to specific habitat requirements and pollinator pressure (Figs S1, S3 and S4 in Additional file 2). It is worth noting that Chen et al. obtained similar phylogenetic results [59]. The authors point out the similarity to *D*. *hekounse*, but we think that *D*. *hekounse* is also morphologically different from other members of the nominal section. Despite the phylogenetic affiliation, *D*. *zhenghuoense* has a clearly different set of morphological characters and in our opinion does not belong to the section *Dendrobium*.

Working on a genus as complex as *Dendrobium* requires comparing data from as many sources as possible. When we analyze the results, it is easy to see that some differences or similarities often overlap and do not provide clear answers. Our studies show that the morphological similarity analysis of both vegetative and floral characteristics is relatively consistent in the case of less representative sections. On the other hand, numerous representative sections, such as the nominal one and closely related, e.g. *Holochrysa* or *Breviflores,* are already more morphologically variable, especially in the floral characters. This may be related to the potential pollinator. Phylogenetically, however, they form a fairly coherent and homogeneous group, with a few exceptions such as *Flickingeria bicolor*, *D*. *khasianum*, or *D*. *calocephalum*. This makes it more difficult to delineate between them, or, as Schuiteman have suggested, to tend to group them together [2]. There is such a remarkable similarity in the entire nominal section, especially between the floral patterns that are observed. But we found some arguments that led us to include or exclude some species from this section. Currently, two extreme approaches to defining taxonomic units of generic rank can be observed. The first approach involves combining all taxa with a monophyletic origin into a single taxon—essentially a supergenus—even if they exhibit structural discontinuities, such as morphological differences. Examples from recent years include *Maxillaria* [60], *Bifrenaria* [61], *Herminium* [62, 63], and *Oncidium* [64, 65]. In such cases, the species range corresponds to previously defined subtribes. While this method produces monophyletic groups, the challenge of internal classification is deferred to the intrageneric level. The second approach focuses on identifying groups that are not only monophyletic but also exhibit distinct and unique combinations of morphological traits. This approach is more challenging as it requires not only the analysis of selected genetic markers but also a detailed examination of the morphological and anatomical structures of the species being studied. *Dendrobium* is an excellent illustration of both approaches to genus definition and taxonomy. Schuitemann proposes a broad view of the genus, which includes all genera of the subtribe Dendrobiinae [2]. On the other hand, Clements [34] and Clements & Jones [33] divided *Dendrobium* into as many as 3 subtribes and a total of 50 genera. In our opinion, *Dendrobium* should be broken down into several genera, but the proposals of the Australian researchers are premature. As our study shows, most of the genera they distinguish are well nested in other *Dendrobium* clades or show polyphyletism. Our results clearly show that *Dendrobium *sensu lato is a genus with an intricate history in which it is possible to observe different rates of evolution, the loss of certain characteristics and the appearance of others in the process of migration to new areas and under the influence of adaptation to local pollinators, in which episodes of hybridization have also occurred. The taxonomy of such a large, diverse, wide-ranging group is and must be complex. Therefore, many years of intensive research are still ahead of us to find the key to total evidence taxonomy.

## Conclusions

Results of our analyses show that *Dendrobium *sensu lato is likely to have undergone convergent evolution. Many floral features have evolved multiple times independently. This leads to numerous classification problems within the genus. We did not get a clear answer on how to classify species within the entire genus. For this reason, in this study we focused mainly on the nominal section. Thus, we treat the determination of species within the nominal section as a priority. It is worth keeping in it only those species that, based on their set of characters, leave no doubt about their membership. Because of such a large group, it is best to take small steps, analyze individual sections, and then put all the data together. Otherwise, studying the entire genus may cause many problems and ambiguities.

Convergent evolution can be the result of adaptation to pollinators or, more commonly, the result of hybridization. This is a common phenomenon in plants, making it difficult to establish a consistent classification system, especially for a genus as numerous and diverse as *Dendrobium*. In this article, we have not performed analyses that could clearly confirm or refute this theory. However, it is worth paying special attention to this issue in the future.

## Supplementary Information

Additional file 1. A list of GenBank ID numbers. Table S1. List of taxa used in the molecular study for ITS marker including GenBank Accession Number.

Additional file 2. Ancestral state reconstruction of morphological features. Table S2. Data matrix used for ancestral state reconstruction of morphological features, where taxa characters were coded for the presence (1, 2 - yes) or absence (0 - no) of a feature. Fig. S1. Ancestral state reconstruction of morphological characters (pseudobulbs) of Dendrobium taxa (according to Table S2). Fig. S2. Ancestral state reconstruction of morphological characters (leaves) of Dendrobium taxa (according to Table S2). Fig. S3. Ancestral state reconstruction of morphological characters (inflorescence) of Dendrobium taxa (according to Table S2). Fig. S4. Ancestral state reconstruction of morphological characters (lip and mentum) of Dendrobium taxa (according to Table S2).Circles next to taxon names indicate geographic distribution.

Additional file 3. Morphological similarities. Table S3. List of morphological characters used to describe Dendrobium sensu lato species included in the cluster and multivariate analyses. Table S4. Data matrix of 14 morphological traits used in the analysis of morphological variation in Dendrobium sensu lato. The data were transformed according to Table S3 in this supplementary file. Table S5. SIMPER analysis identifying the percentage of similarity and dissimilarity of all morphological traits studied (A) and only floral traits (B) for each section of Dendrobium sensu lato. For a detailed description of morphological characters is provided in Table S3 in this supplementary file. Fig. S5. Two-way UPGMA cluster analysis based on Jaccard similarity coefficients, showing morphological relationships within Dendrobium sensu lato, with regard to the division into sections. A detailed description of the morphological characters can be found in Table S3 in the additional file. Fig. S6. Cluster analysis performed using Ward's method, showing similarity with respect to floral traits for Dendrobium sensu lato species. The clusters and subclusters that are the subject of discussion are indicated by upper and lower case letters and numbers. A detailed description of the floral traits can be found in Table S3 in Additional file 3. Fig. S7. Non-metric multidimensional scaling analysis, NMDS (A) and principal coordinate analysis, PCoA (B) showing the two-dimensional ordering of Dendrobium sensu lato specimens, based on only 5 floral characters. Convex hulls for each Dendrobium section were added to the plots.

Additional file 4. Summary of the taxonomy of the Dendrobium section. Table S6. Nominal section species according to various authors and the results obtained.

## Data Availability

The DNA sequences obtained in this study have been deposited in the NCBI database (https://www.ncbi.nlm.nih.gov/genbank/). The accession numbers are provided in the supplementary information. All data generated or analyzed during this study are included in this published article and its supplementary information files.
