# Supplementary material for: Evolution of morphological traits of Dendrobium sensu lato (Orchidaceae)—an attempt to resolve phylogenetic relationships in nominal and morphologically convergent sections
Source: BMC Plant Biol. 2025 Feb 22;25:239. doi: 10.1186/s12870-025-06263-w (PMC11846450; doi:10.1186/s12870-025-06263-w)
Supplement: Supplementary file 2 — Additional file 2. Ancestral state reconstruction of morphological features. Table S2. Data matrix used for ancestral state reconstruction of morphological features, where taxa characters were coded for the presence (1, 2 - yes) or absence (0 - no) of a feature. Fig. S1. Ancestral state reconstruction of morphological characters (pseudobulbs) of Dendrobium taxa (according to Table S2). Fig. S2. Ancestral state reconstruction of morphological characters (leaves) of Dendrobium taxa (according to Table S2). Fig. S3. Ancestral state reconstruction of morphological characters (inflorescence) of Dendrobium taxa (according to Table S2). Fig. S4. Ancestral state reconstruction of morphological characters (lip and mentum) of Dendrobium taxa (according to Table S2).Circles next to taxon names indicate geographic distribution. [file 12870_2025_6263_MOESM2_ESM.zip › Additional file 2. Fig. S1.pdf]

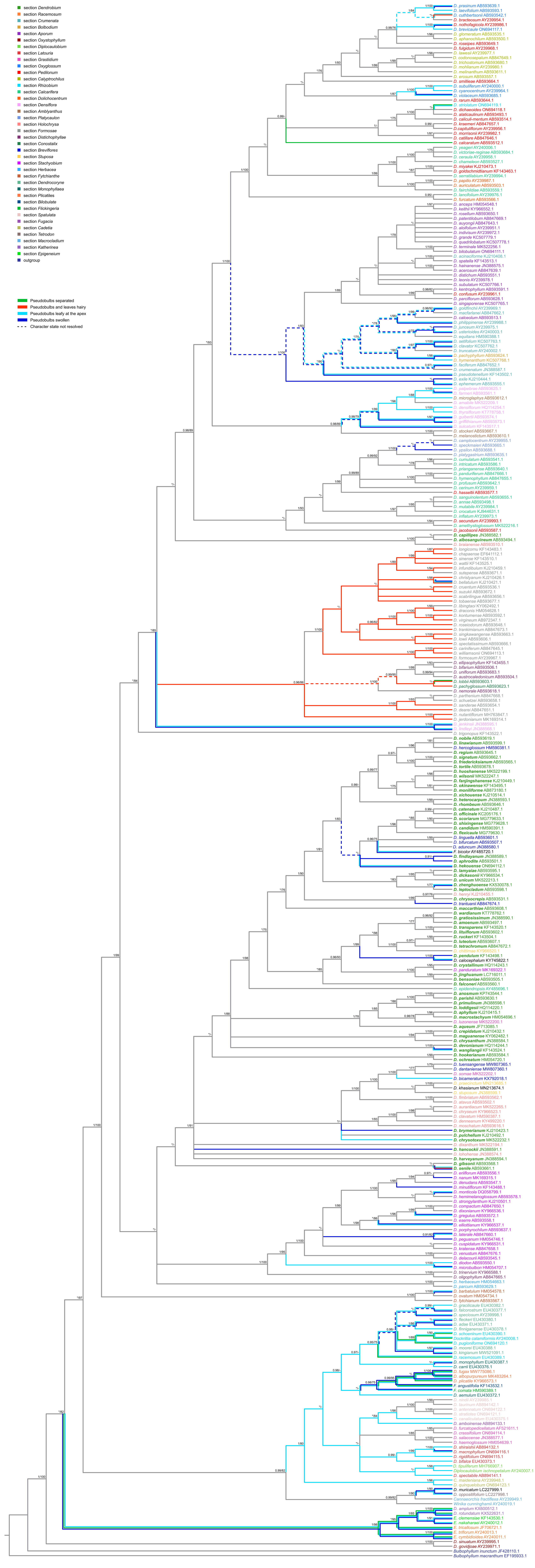

Fig. S1. Ancestral state reconstruction of morphological characters (pseudobulbs) of *Dendrobium* taxa (according to Table S2).
