# Supplementary material for: Evolution of morphological traits of Dendrobium sensu lato (Orchidaceae)—an attempt to resolve phylogenetic relationships in nominal and morphologically convergent sections
Source: BMC Plant Biol. 2025 Feb 22;25:239. doi: 10.1186/s12870-025-06263-w (PMC11846450; doi:10.1186/s12870-025-06263-w)
Supplement: Supplementary file 3 — Additional file 3. Morphological similarities. Table S3. List of morphological characters used to describe Dendrobium sensu lato species included in the cluster and multivariate analyses. Table S4. Data matrix of 14 morphological traits used in the analysis of morphological variation in Dendrobium sensu lato. The data were transformed according to Table S3 in this supplementary file. Table S5. SIMPER analysis identifying the percentage of similarity and dissimilarity of all morphological traits studied (A) and only floral traits (B) for each section of Dendrobium sensu lato. For a detailed description of morphological characters is provided in Table S3 in this supplementary file. Fig. S5. Two-way UPGMA cluster analysis based on Jaccard similarity coefficients, showing morphological relationships within Dendrobium sensu lato, with regard to the division into sections. A detailed description of the morphological characters can be found in Table S3 in the additional file. Fig. S6. Cluster analysis performed using Ward's method, showing similarity with respect to floral traits for Dendrobium sensu lato species. The clusters and subclusters that are the subject of discussion are indicated by upper and lower case letters and numbers. A detailed description of the floral traits can be found in Table S3 in Additional file 3. Fig. S7. Non-metric multidimensional scaling analysis, NMDS (A) and principal coordinate analysis, PCoA (B) showing the two-dimensional ordering of Dendrobium sensu lato specimens, based on only 5 floral characters. Convex hulls for each Dendrobium section were added to the plots. [file 12870_2025_6263_MOESM3_ESM.zip › Additional file 3. Fig. S5.pdf]

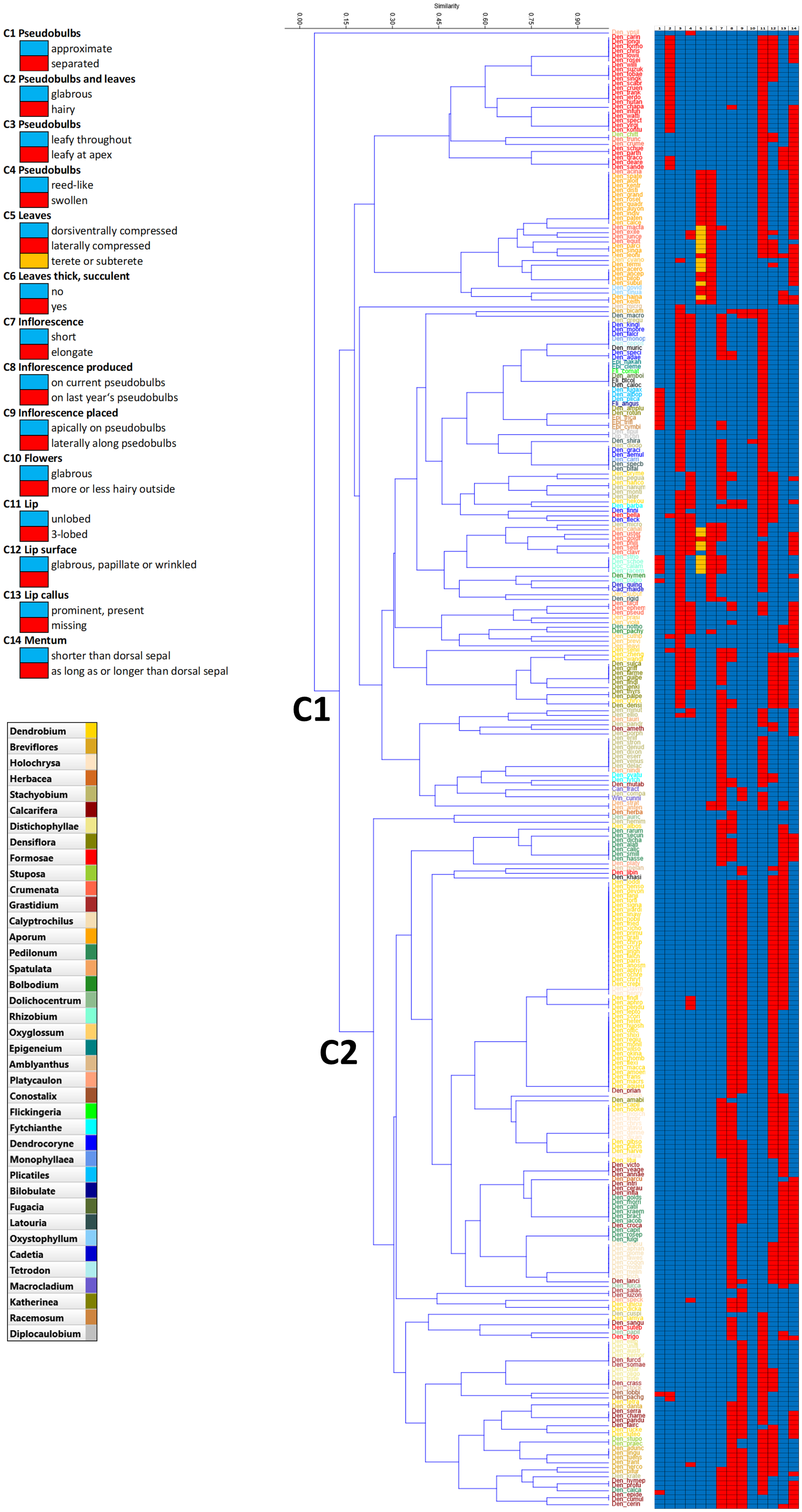

Fig. S5. Two-way UPGMA cluster analysis based on Jaccard similarity coefficients, showing morphological relationships within *Dendrobium sensu lato*, with regard to the division into sections. A detailed description of the morphological characters can be found in Table S3 in the additional file.
